# Effects of carbohydrate combined with caffeine on repeated sprint cycling and agility performance in female athletes

**DOI:** 10.1186/1550-2783-11-17

**Published:** 2014-05-01

**Authors:** Chia-Lun Lee, Ching-Feng Cheng, Todd A Astorino, Chia-Jung Lee, Hsin-Wei Huang, Wen-Dien Chang

**Affiliations:** 1 Physical Education Section of General Education, National Sun Yat-sen University, Kaohsiung, Taiwan; 2 Department of Athletic Performance, National Taiwan Normal University, Taipei, Taiwan; 3 Department of Kinesiology, California State University-San Marcos, San Marcos, CA, USA; 4 Department of Physical Education, National Taiwan Normal University, Taipei, Taiwan; 5 Department of Sports Medicine, China Medical University, Taichung, Taiwan

**Keywords:** Anaerobic capacity, Ergogenic aids, Fatigue, Hormone, Metabolic substrate, Nutrition

## Abstract

**Background:**

Caffeine (CAF) has been shown to improve performance during early phase of repeated sprint exercise; however some studies show that CAF also increases the magnitude of physical stress represented by augmented blood lactate, glucose, and cortisol concentrations during latter phase of repeated sprint exercise. No studies have investigated the efficacy of combined carbohydrate (CHO) and CAF consumption during repeated sprint exercise (RSE) in female athletes. Thus, the purpose of this study was to investigate the effects of CAF with CHO supplementation on RSE and agility.

**Methods:**

Eleven female athletes completed four experimental trials performed 7 d apart in a double-blind, randomized, and counter-balanced crossover design. Treatments included CAF + PLA (placebo), CAF + CHO, PLA + CHO, and PLA + PLA. Participants ingested capsules containing 6 mg · kg^−1^ of CAF or PLA 60-min prior to RSE, and 0.8 g · kg^−1^ of CHO solution or PLA immediately before the RSE, which consisted of ten sets of 5 × 4-s sprints on the cycle ergometer with 20-s active recovery. The agility T-test (*AT-test*) was performed before and after the RSE. Blood samples were acquired to assess glucose, lactate, testosterone, and cortisol.

**Results:**

During Set 6 of RSE, peak power and mean power were significantly higher in PLA + CHO than those in CAF + PLA and PLA + PLA, respectively (*p* < .05). Total work was significantly increased by 4.8% and 5.9% with PLA + CHO than those of CAF + CHO and CAF + PLA during Set 3. PLA + CHO also increased total work more than CAF + PLA and PLA + PLA did during Set 6 (*p* < .05). No significant differences in *AT-test* performance either before or after the RSE were occurred among treatments (*p* > .05). Blood lactate and glucose concentrations were significantly higher under CAF + CHO, CAF + PLA, and PLA + CHO versus PLA + PLA (*p* < .05), but no differences in testosterone or cortisol levels were found (*p* > .05).

**Conclusions:**

Findings indicate that CAF + PLA or CAF + CHO ingestion did not improve repeated sprint performance with short rest intervals or agility. However, CHO ingested immediately prior to exercise provided a small but significant benefit on RSE performance in female athletes.

## Background

Most team sports include performance of moderate- to long duration exercise interspersed with repeated bouts of high-intensity activities as well as periods of low-to-moderate active recovery or passive rest. The work: rest ratio of the team sport athlete is around 1:4.5 [1], and average number of sprints completed during competition is approximately 20–60 times with an approximate sprint duration equal to 2 – 4-s [2]. Girard et al. [3] reported that intermittent sprint exercise (ISE) differs greatly from repeated sprint exercise (RSE), that is, ISE is characterized by short-duration sprints (≤10-s) interspersed with long recovery periods (60–300-s); however, RSE is characterized by similar exercise duration (≤10-s) interspersed with insufficient recovery (≤60-s). Gaitanos et al. [4] indicated that the inadequate recovery inherent in RSE (6-s maximal sprints with 30-s rest intervals) may impair sprint performance because of limited adenosine triphosphate (ATP) supply from anaerobic metabolism (glycolysis and phosphocreatine (PCr) resynthesis) during the transient recovery between sprints, and increased acidosis. Thus, the strategies of nutritional ingestion are needed to preserve repeated sprint performance in competitive athletes.

It is common practice for team sport athletes to consume carbohydrate (CHO) to improve intermittent exercise capacity [5,6] and endurance performance [7,8], which is thought to occur via central nervous system (CNS) activation and other potential mechanisms such as higher rates of CHO oxidation [9,10]. Another ergogenic aid that has routinely been used by athletes is caffeine (CAF) [11]. Existing data show that CAF supplementation may benefit sprint performance [12,13] and reactive agility performance [14] via various mechanisms [15]. However, one study demonstrated that caffeine was ergolytic for mean power and fatigue index during the high-intensity sprint test when a 24 × 4-s cycling sprint test with 20-s of active recovery was completed versus a 90-s active recovery between each sprint bout [16]. Numerous studies have also reported that CAF ingestion has a small or negligible effect on sprint performance [16-18] when repeated sprint tests (≤10-s) are interspersed with short rest periods (≤60-s), as well as no effect on reactive agility [19]. Although CAF significantly improved ISE [12,13,20], a number of studies have suggested that CAF doses of 2–6 mg · kg^−1^ are likely to improve ISE but not RSE performance; in other words, caffeine ingestion may negatively affect repeated sprint performance with short recovery intervals in the later stages of exercise [16,21]. If CHO plus CAF could potentiate benefits of CHO on substrate metabolism and improve CNS modulation, then CAF may enhance RSE performance. Some studies have examined changes in metabolism when CAF is coingested with CHO. For example, Yeo et al. [22] found that coingestion of CHO with CAF promoted intestinal glucose absorption resulting in greater exogenous CHO oxidation than CHO ingestion alone. In addition, intestinal glucose absorption was significantly increased with carbohydrate-electrolyte plus CAF compared with a carbohydrate-electrolyte solution alone [23]. Several studies show that combined intake of CHO and CAF may be ergogenic for intermittent sprint performance later in exercise [24-27] and lower rating of perceived exertion (RPE) and fatigue index [28]. However, certain studies have reported that ingesting CHO with CAF does not affect time-trial performance [23,29,30]. Thus, further studies are needed to clarify the effects of CHO and CAF coingestion on RSE performance.

Team sports require many skills other than running in a straight line, including brief pauses, cutting actions, and rapid direction and speed changes, which all are important elements of agility. The consequences of studies focused on the improvements of agility performance after ingesting CAF and/or CHO remain controversial. Duvnjak-Zaknich et al. [14] showed that ingesting CAF may benefit reactive agility in trained male athletes, but Lorino et al. [19] indicated that CAF does not improve proagility shuttle run performance in young adult males. Roberts et al. [25] investigated the combined effects of CHO and CAF on a sustained high-intensity test of speed and agility in male rugby players, indicating the agility performance was not significantly different between trials but the likelihood of 2% improvements for CHO + CAF over placebo. In female soccer players, Red Bull containing low doses of CAF (80 mg; ~ 1.3 mg · kg^−1^) and CHO (27 g; ~ 0.4 g · kg^−1^) did not provide ergogenic effects on repeated agility T-test performance [31]. However, there are limited evidences investigating the effects of CHO and/or CAF with moderate dosage on agility performance in female athletes. It is unclear whether CAF or CHO + CAF supplementation by female athletes, especially in team sports, enhances agility in change of direction (e.g. agility T-test) and in fatigued condition (e.g. after a long-time repeated sprint test rather than short-time). Thus, further studies should be conducted to clarify the effects of CAF and/or CHO supplementation on agility performance during various exercise stages.

Although no significant differences were found on salivary testosterone and cortisol concentrations after repeated bouts of supra-maximal exercise in female adolescents [32], ingestion of CAF with moderate dose might elevate the salivary cortisol concentrations [33], and the benefit of caffeine on performance might be counteracted by the increases in cortisol and the decreases in testosterone: cortisol ratio [34]. Walker et al. [35] reported that ingesting a placebo and CAF increased cortisol concentration more than ingesting only CHO after a 2-h endurance cycling exercise. CHO could offer some protection against the fall in testosterone: cortisol ratio during short-term intense exercise training [36]. It is likely that the effects of CHO on cortisol release regulation are larger than CAF, and may occur by activating the hypothalamic-pituitary-adrenal axis, providing a natural negative-feedback system through the coordination of cortisol, whereas no effect has been observed on hormonal and physiological responses after RSE [37]. However, it is unclear whether ingesting CHO, or CAF and/or CHO causes RSE performance changes and hormonal reactions in women.

To date, no study examined the effect of ingestion of caffeine + placebo (CAF + PLA), caffeine + carbohydrate (CAF + CHO), carbohydrate + placebo (CHO + PLA), or placebo + placebo (PLA + PLA) on prolonged period of repeated sprint ability and agility performance for women in team sports. Therefore, the primary purpose of this study was to examine the effects of ingesting CAF combined with PLA, CAF + CHO, CHO + PLA, or PLA + PLA on repeated sprint performance tasks simulating team sports in female athletes. It is hypothesized that (1) CAF + CHO may improve repeated sprint performance and agility more than CAF + PLA and PLA + PLA do, and (2) CAF + PLA or CAF + CHO may affect blood metabolism throughout repeated sprint exercise (RSE).

## Methods

### Participants

Eleven trained female athletes (age = 21.3 ± 1.2 yr, height = 164.2 ± 5.7 cm, and body mass = 58.6 ± 7.3 kg), members of Division I collegiate team-sport teams, volunteered to take part in this study. They reported habitual caffeine intake = 50 to 100 mg · d^−1^. All participants were regularly involved in team-sport competition such as basketball or volleyball and engaged in training 12.6 ± 1.2 hours/week. Participants were informed of the experimental procedures and potential risks before providing written informed consent. Prior to a familiarization session replicating the experimental procedure, all participants were screened for medical history and legal ergogenic aids use, and the results showed that none had taken any medicines (included prescription and over-the-counter medications) or ergogenic aids (which may influence multiple sprint performance, e.g., creatine) for at least 3 months prior to the experiment. A comprehensive list of dietary food products and medicines containing caffeine was provided to participants prior to the first familiarization trial. Participants abstained from all foods and liquids containing caffeine for 48-h before the experimental trials, as well as any alcohol and intense exercise for at least 24-h prior to all sessions. In addition, participants completed a questionnaire inquiring whether they experienced nausea, vomiting, muscle cramps, flatulence, diarrhea, anxiety, quivering, headaches, or other symptoms in order to evaluate any side effects experienced prior to exercise testing. The investigation was approved by the University Institutional Review Board.

### Experimental design

**Figure 1:**
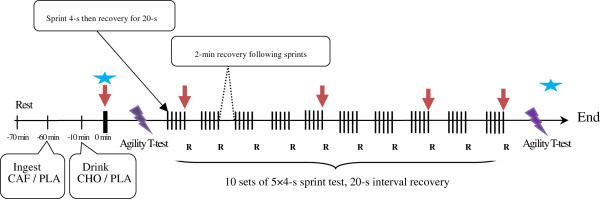
**Schematic diagram of the 10 sets of 5 × 4-s repeated sprint cycling test.** ↓: blood lactate and glucose. CAF: caffeine trial; PLA: placebo trial; CHO: carbohydrate trial. Asterisk: cortisol and testosterone. Lightning: agility T-test. R: rating of perceived exertion.

Each participant visited the laboratory on five separate occasions. The first visit included preliminary testing to familiarize participants with the procedures and to minimize any learning effects. Once familiar with the protocol, each participant undertook four experimental trials separated by at least 7 d. Treatment order was randomly assigned and counterbalanced using a Latin squares design, and was provided in a double-blind fashion, participants and researchers were blind to treatment assignment. After ingestion, the participants completed the agility T-test (*AT-test*) and RSE after a dynamic warm up. The *AT-test* used in this study was similar with a previous study that showed this test has a highly reliability and validity [38]. During exercise, heart rate (HR) was regularly assessed with a Polar heart rate monitor (Polar S810i™, Polar Electro Inc, Finland) and the RPE was measured using a Borg 6–20 RPE scale [39]. Participants were familiarized with the RPE scale during the preliminary test. Blood samples were obtained throughout exercise (Figure 1).

### Treatment ingestion

Participants completed four experimental trials: CAF + PLA, CAF + CHO, CHO + PLA, and PLA + PLA. Participants arrived at the laboratory according to the time sheet. Within subjects, the time of each trial remained consistent for all trials to avoid any influence of circadian variance. On arrival to the laboratory, participants were provided with a prepacked meal with an energy content of 492.75 Kcal, composed of 64% carbohydrate, 23% fat, and 13% protein. At 7:00 AM, after consuming their prepacked breakfast, participants ingested opaque gelatin capsules containing either 6 mg · kg^−1^ of CAF (Sigma-Aldrich, Sydney, Australia) or an equal dosage of placebo (cellulose, Holy Food, Taoyuan, Taiwan), along with 200 ml of water [16]. Participants then rested in a quiet room for 50-min prior to ingesting the carbohydrate solution drink or placebo. Before commencing the agility and repeated sprint exercise, participants were asked to describe onset of symptoms or side effects from caffeine ingestion; thereafter, participants consumed either a CHO solution containing 0.8 g · kg^−1^ body mass dextrose (Roquette, France) with 500 ml of orange-flavored water or a placebo consisting of low-calorie artificial sweetener (Prinsen BV, Helmond, The Netherlands) with 500 ml of flavored water, and then participants consumed 300–500 ml water throughout the testing. The appearance and taste of solutions were similar among treatments.

### Agility T-test (*AT-test*)

The *AT-test*, referred to a previous study [38], was performed before and after the RSE. This protocol has been used to assess the agility of athletes participating in team-sport exercise [40,41]. It is a highly reliable measure of leg speed, leg power, and agility [38]. The agility test requires participants to run forward, lateral, and backward, as quickly as possible, and the total distance is 40 yard (36.56 m). Each trial was timed from start to completion by using an electronic timing system (Smart-Speed, Fusion Sport, Australia). Speed decrement of the *AT-test* was calculated based on a previous study [42]. The intra-class correlation coefficient (ICC, 0.87-0.98) and the coefficient of variance (CV, 4.3%-4.6%), which was calculated from the data between familiarization trial and first bout of *AT-test* in PLA + PLA trial, was good for *AT-test*.

### Repeated sprint test

Participants were weighed to determine the accurate load for the RSE, which was performed on a cycle ergometer (Avantronic Cyclus II, h/p Cosmos®, Germany). The predetermined resistance was calculated according to body mass by using the following equation, produced by internal software: 0.7 × body mass in kg/0.173. Then, participants performed a standardized warm up followed by the first T test. A brief unloaded sprint allowed participants to prepare for the subsequent RSE. Participants were required to stay seated on the cycle ergometer for the entire duration of the RSE to limit the recruitment of other muscle groups. During each sprint, participants were encouraged to cycle maximally for each 4-s bout and pedal as fast as possible against the given load. The protocol for the RSE consisted of ten sets of repeated sprints with 2-min recovery at 50 watts at a self-selected speed (Figure 1). Each set was composed of 5 × 4-s sprints with a 20-s active recovery (60–70 rpm, 50 watts) performed between each sprint. This test was used in a previous study [16] and is designed to activate glycolysis and maximize PCr degradation [2,4]. They were informed at the end of the recovery phase at least 5-s prior to the beginning of the next sprint. Participants were given consistent verbal encouragement during each sprint, but no performance information was provided. The power output data were recorded during each sprint using the cycle ergometer software. After completing the protocol, all data were then transferred to a personal computer to calculate the peak power, mean power, total work, and sprint decrement (equation 1) as used in previous studies [3,42]. The ICC and CV for peak power during RSE were 0.86 - 0.99 and 5.6% - 6.4%, respectively.

(1) $$S_{\mathrm{decrement}}\left(\%\right)=\left\{1-\frac{\left(S1+S2+S3+\ldots +\mathrm{Sfinal}\right)}{\mathrm{Sbest}\times \mathrm{number}\;\mathrm{of}\;\mathrm{sprints}}\right\}\times 100$$

### Blood analysis

Blood samples (5 mL) were drawn with an indwelling venous cannula following treatment ingestion and immediately after exercise testing. This sample was placed in a tube and centrifuged at 3000 rpm for 15-min. The resultant serum was stored at −80°C for subsequent analysis of concentrations of cortisol and testosterone using radioimmunoassay (Wizard^2^ Automatic Gamma Counter, PerKin-Elmer Corp, USA), with a CV of less than 5% according to LEZEN reference laboratory (Taipei, Taiwan). In addition, a 20 μl blood sample for analyzing blood glucose and lactate concentrations was collected from the earlobe immediately before RSE exercise (i.e. pre-test, which means the time point at 10 min after drinking CHO/PLA beverage), and after sets 1, 5, 8, and 10 of RSE exercise. To assess changes in blood glucose, a 10 μl earlobe blood sample was analyzed by Byer analyzer (Ascencia Breeze, Bayer HealthCare LLC, USA), and the remaining blood sample was used to obtain blood lactate concentration using methods described previously [16].

### Statistical analyses

Data are reported as mean ± standard deviation and were analyzed with SPSS for Windows (version 17.0, SPSS, Inc., Chicago IL, USA). Dependent variables (peak power, mean power, total work, and RPE) were analyzed using a ten (numbers of set) by four (treatment: CAF + PLA, CAF + CHO, PLA + CHO, and PLA + PLA), two-way repeated-measures analysis of variance (ANOVA). Changes in concentration of lactate, glucose, cortisol, and testosterone as well as agility performance between treatments and over time were also analyzed with two-way repeated-measures ANOVA. One-way ANOVA was performed to study differences in performance decrement of *AT-test* and RSE between treatments. To minimize the violation of the assumption of homogeneity of variance, the Greenhouse-Geisser correction was used when sphericity was violated. When differences were identified by ANOVA, the Bonferroni adjustment was used to ascertain where the differences lay. Statistical significance was set at a *p* value of ≤ .05 for all analyses. The ICC and CV were computed from the data between familiarization and PLA + PLA trials to determine the test**-**retest reliability of the RSE and *AT-test*. Effect size was expressed as partial eta squared (η^2^). According to Portney et al. [43] , the magnitude of difference in key dependent variables is expressed as the η^2^ using the following criteria: small η^2^ = .01, medium η^2^ = .06, large η^2^ = .14.

## Results

### Repeated sprint ability

#### Peak power

**Figure 2:**
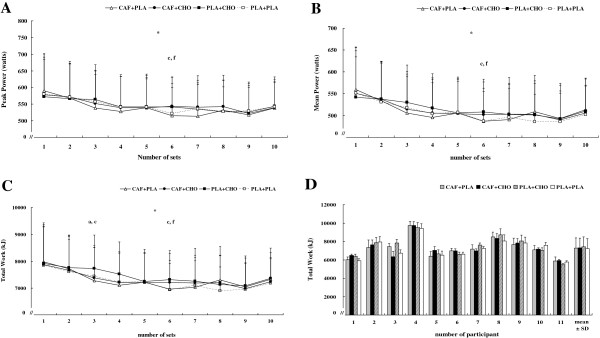
**Changes in peak power (A), mean power (B), and total work (C) for each set of the repeated sprint test (10 sets of 5 × 4-s sprint with 20-s of rest intervals; 2-min recovery after each set) for the conditions of caffeine + placebo (CAF + PLA), caffeine + carbohydrate (CAF + CHO), placebo + carbohydrate (PLA + CHO), and placebo + placebo (PLA + PLA).** Individual differences in total work **(D)** for each condition throughout the testing. * = significant time effect (*p* < .05). a = significant difference between CAF + CHO and PLA + CHO (*p* < .05). c = significant difference between CAF + PLA and PLA + CHO (*p* < .05). f = significant difference between PLA + CHO and PLA + PLA (*p* < .05). Values are mean ± standard deviation.

There was a significant interaction for peak power (*F* = 1.89, η^*2*^ = 0.16, *p* < .01). Figure 2A shows a significant difference in peak power output between PLA + CHO and CAF + PLA (*p* < .05). Additionally, there was a significant difference in peak power across bouts among all treatments, as it declined across bouts. A main treatment effect was observed in Set 6 (*F* = 5.02, η^*2*^ = 0.33, *p* < .01); *post hoc* analyses revealed there was a trend for greater peak power (+3.8%) in PLA + CHO than PLA + PLA (*p* = .08) and in CAF + CHO than CAF + PLA (+5.3%) (*p* = .08), respectively; however, this difference was non-significant.

#### Mean power

Figure 2B summarizes changes in mean power during the RSE for each treatment. There was a significant treatment × time interaction for mean power (*F* = 1.64, η^*2*^ = 0.14, *p* < .05). In PLA + CHO, mean power differed from PLA + PLA at set 6 of RSE (*p* < .05), but no difference was observed between CAF + PLA, CAF + CHO, PLA + CHO, and PLA + PLA across all other sets (*p* > .05). Mean power was higher in set 1 than subsequent sprint sets across all treatments (*p* < .05).

#### Total work

There was a significant treatment × time interaction for total work (*F* = 1.64, η^*2*^ = 0.03, *p* < .05). Compared with the PLA + PLA condition, total work in set 6 of PLA + CHO was significantly increased by 5.2% (*F* = 3.20, η^*2*^ = 0.24, *p* < .05) and greater by 4.1% (*F* = 3.26, η^*2*^ = 0.25, *p* < .05) versus CAF + PLA during RSE; however, total work with CAF + CHO did not differ from CAF + PLA or PLA + PLA in any of the other sets (*p* > .05) (Figure 2C). Total work declined across sets in all treatments (*p* < .01). Individual responses in total work are shown in Figure 2D. Most participants expressed minimal changes in work, although subject 3 revealed lower performance after CAF + CHO supplementation.

#### RSE decrement, HR, and RPE

**Figure 3:**
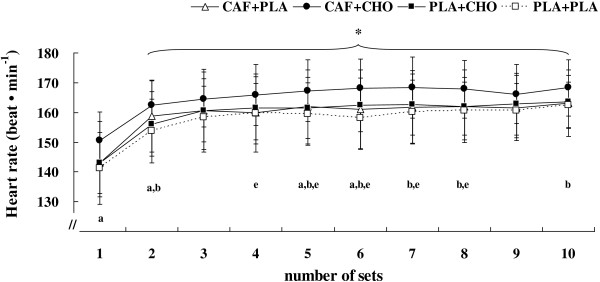
**Change in heart rate during each set of the repeated sprint test for the conditions of caffeine + placebo (CAF + PLA), caffeine + carbohydrate (CAF + CHO), placebo + carbohydrate (PLA + CHO), and placebo + placebo (PLA + PLA).** * = significant time effect (*p* < .01). a = significant difference between CAF + CHO and PLA + CHO (*p* < .05). b = significant difference between CAF + CHO and PLA + PLA (*p* < .05). e = significant difference between CAF + PLA and PLA + CHO (*p* < .05). Values are mean ± standard deviation.

**Figure 4:**
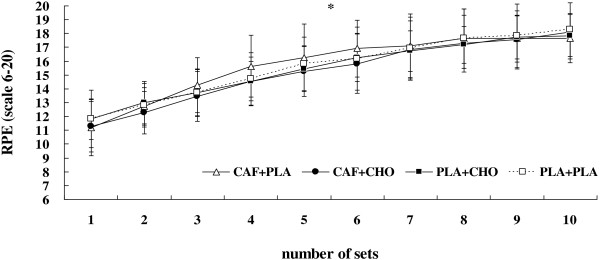
**Change in rating of perceived exertion (RPE) during each set of the repeated sprint test for the conditions of caffeine + placebo (CAF + PLA), caffeine + carbohydrate (CAF + CHO), placebo + carbohydrate (PLA + CHO), and placebo + placebo (PLA + PLA).** * = significant time effect (*p* < .01). *Post hoc* analyses show no significant difference was observed between treatments in any of the other sets (*p* > .05). Values are mean ± standard deviation.

Sprint decrement in total work was not significantly different between CAF + PLA (18.5 ± 5.5%), CAF + CHO (15.5 ± 4.6%), PLA + CHO (16.2 ± 4.3%), or PLA + PLA (17.3 ± 2.8%) (*F* = 1.33, η^*2*^ = 0.12, *p* > .05). As shown in Figure 3, average HR during each set of the RSE was significantly higher in CAF + CHO compared with CAF + PLA, PLA + CHO, and PLA + PLA (*F* = 7.76, η^*2*^ = 0.44, *p* < .01). There was a significant change in HR across sets (*F* = 80.49, η^*2*^ = 0.89, *p* < .01), as HR increased from values equal to 144.5 ± 3.0 beats/min (95% CI = 137.9 ± 151.1 beats/min) from set 1 to near 164.4 ± 3 beats/min (95% CI = 158.7 ± 170.2 beats/min) at set 10. However, no interaction was revealed for heart rate (*F* = 0.97, η^*2*^ = 0.09, *p* > .05). In addition, there was no significant treatment × time interaction for RPE during the RSE (*F* = 1.55, η^*2*^ = 0.13, *p* > .05), whereas, RPE significantly increased during RSE in all treatments (*p* < .05) (Figure 4).

#### Blood lactate and glucose concentrations

**Figure 5:**
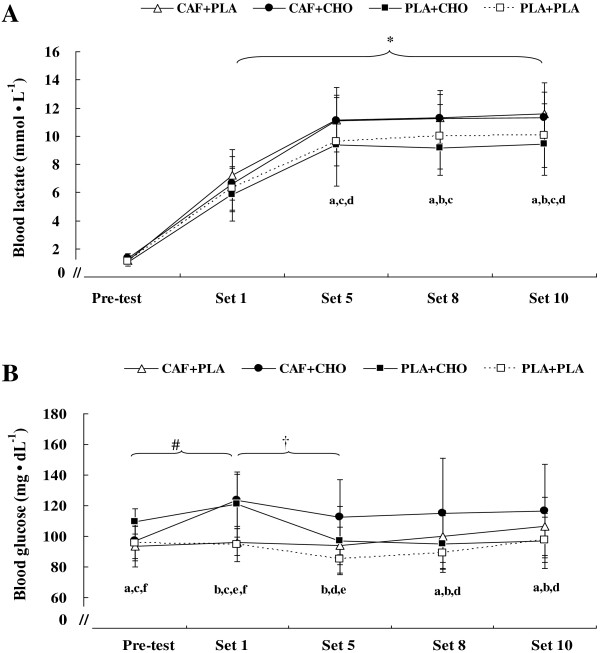
**Changes in blood lactate (A) and glucose (B) concentration at pre-test and after set 1, 5, 8, and 10 for the conditions of caffeine + placebo (CAF + PLA), caffeine + carbohydrate (CAF + CHO), placebo + carbohydrate (PLA + CHO), and placebo + placebo (PLA + PLA).** * = significant increase from pre-test (*p* < .01). # = significant increase from pre-test in the CAF + CHO (*p* < .05). † = significant decrease from set 1 in the PLA + CHO (*p* < .05). a = significant difference between CAF + CHO and PLA + CHO (*p* < .05). b = significant difference between CAF + CHO and PLA + PLA (*p* < .05). c = significant difference between CAF + PLA and PLA + CHO (*p* < .05). d = significant difference between CAF + PLA and PLA + PLA (*p* < .05). e = significant difference between CAF + PLA and PLA + CHO (*p* < .05). f = significant difference between PLA + CHO and PLA + PLA (*p* < .05). Values are mean ± standard deviation.

There was a main effect for time and treatment (*p* < .01) as well as an interaction for blood lactate concentration during exercise (*F* = 2.57, η^*2*^ = 0.20, *p* < .01). Post hoc analyses show that blood lactate concentrations in CAF + PLA and CAF + CHO conditions were significantly higher than those in PLA + CHO and PLA + PLA conditions for Sets 5, 8, and 10 throughout the RSE (*p* < .05; Figure 5A). Blood lactate concentration increased from Set 1 to the last Set and was significantly higher than pre-test (*p* < .01) in all conditions.

There was an interaction for blood glucose concentration (*F* = 7.53, η^*2*^ = 0.43, *p* < .01) as well as a main effect for treatment and time during exercise. *Post hoc* for treatment shows blood glucose was significantly higher in PLA + CHO compared with other treatments at pre-test and Set 1 during RSE, but caffeine ingestion combined with carbohydrate or placebo significantly increased glucose levels during subsequent RSE (Figure 5B). In addition, *post hoc* analyses show that blood glucose concentration was significantly higher at Set 1 compared to pre-test in CAF + CHO (*p* < .01), and higher blood glucose at Set 1 versus Set 5 in PLA + CHO (*p* < .05). In addition, blood glucose concentration remained stable throughout RSE with CAF + PLA and PLA + PLA ingestion (*p* > .05).

#### Serum cortisol and testosterone concentrations

**Figure 6:**
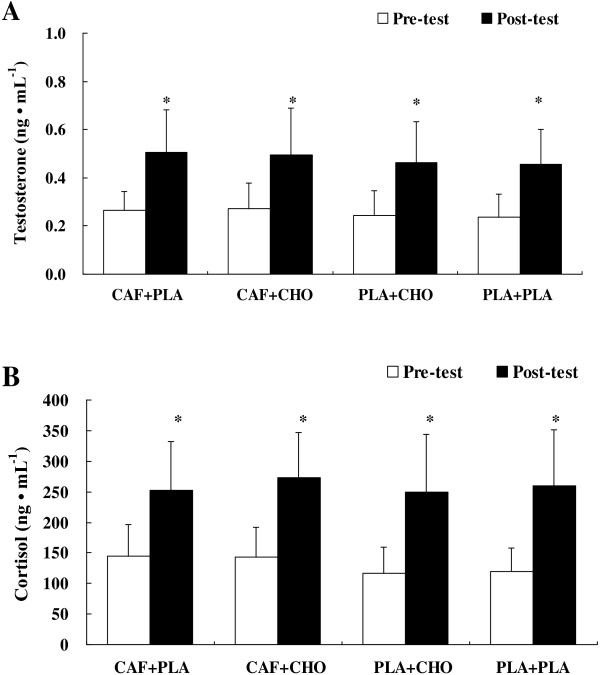
**Changes in serum testosterone (A) and cortisol (B) concentrations in the conditions of caffeine + placebo (CAF + PLA), caffeine + carbohydrate (CAF + CHO), placebo + carbohydrate (PLA + CHO), and placebo + placebo (PLA + PLA).** * = significant increase from pre-test (*p* < .01). Values are mean ± standard deviation.

No significant interaction was observed for serum cortisol (*F* = 0.34, η^*2*^ = 0.33, *p* = .79) or testosterone (*F* = 0.31, η^*2*^ = 0.03, *p* = .59), and there was no treatment effect for serum cortisol (*F* = 0.86, η^*2*^ = 0.08, *p* = .48) or testosterone (*F* = 3.60, η^*2*^ = 0.26, *p* = .09). However, post-exercise serum cortisol and testosterone concentrations were significantly higher than at pre-test in all treatments (*p* < .05) (Figures 6A-B). Additionally, no significant treatment × time interaction (*F* = 0.29, η^*2*^ = 0.03, *p* = .84) or treatment effect were observed in testosterone/cortisol ratio at pre-test (CAF + PLA vs. CAF + CHO vs. PLA + CHO vs. PLA + PLA; 2.04 ± 0.83 vs. 1.93 ± 0.62 vs. 2.12 ± 0.59 vs. 2.24 ± 1.20, *p* > .05) or at post-test (CAF + PLA vs. CAF + CHO vs. PLA + CHO vs. PLA + PLA; 2.03 ± 0.36 vs. 1.90 ± 0.82 vs. 2.00 ± 0.85 vs. 1.91 vs. 0.76, *p* > .05).

#### *AT-test* performance

**Figure 7:**
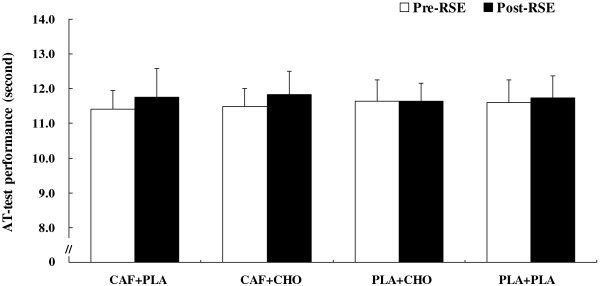
**Changes in agility T-test (*AT-test*) performance for the conditions of caffeine + placebo (CAF + PLA), caffeine + carbohydrate (CAF + CHO), placebo + carbohydrate (PLA + CHO), and placebo + placebo (PLA + PLA).** RSE: repeated sprint exercise. Values are mean ± standard deviation.

The results show that a significant agility performance interaction did not exist (*F* = 2.14, η^*2*^ = 0.18, *p* > .05), as well no significant main effects for time or treatment (Figure 7). Speed decrement was not significantly different among conditions (CAF + PLA vs. CAF + CHO vs. PLA + CHO vs. PLA + PLA, −3.06 ± 5.90% vs. -2.98 ± 3.96% vs. -0.14 ± 2.98% vs. -1.39 ± 4.46%; *F* = 2.14, η^*2*^ = 0.18, *p* > .05). However, agility performance in the PLA + CHO condition was relatively well-preserved compared to the other treatments.

#### Side effects

All participants filled out the side effect questionnaire to assess the possible adverse reaction 60-min after ingesting caffeine or placebo capsule. After ingestion of caffeine, one participant experienced anxiety and slight tremor, another experienced diarrhea, and a third experienced headache and flatulence. However, carbohydrate alone or placebo supplementation did not result in any uncomfortable issues for participants.

## Discussion

To our knowledge, the present study is the first to examine the effects of caffeine (6 mg · kg^−1^) combined with carbohydrate (0.8 g · kg^−1^) administration on repeated sprint performance (10 sets of 5 × 4-s sprint with 20-s rest between each sprint) and agility in female athletes. The main findings indicate a significant increase in peak power, mean power, and total work with carbohydrate ingestion alone prior to commencing a repeated sprint exercise protocol. However, the sprint decrement and agility performance for the CAF + PLA, CAF + CHO, PLA + CHO, and PLA + PLA conditions were not statistically different. Data also demonstrated that either coingestion of CAF and CHO or CAF alone significantly increased heart rate and blood lactate and glucose concentrations during later stages of the RSE, but did not alter testosterone or cortisol levels.

It has been documented that CAF’s influence on anaerobic exercise capacity and agility may depend on the rest: work ratio [11]. Similar to the results of previous studies by Lee et al.[16], Paton et al. [17], and Stuart et al. [21], CAF alone did not improve repeated sprint ability. Thus, while further applied research certainly needs to be done, these results suggest that CAF provides negligible benefit to repeated sprint exercise with insufficient rest interval (work: rest ratio = 1:5).

Although a meta-analysis indicated that CAF + CHO ingestion improved endurance performance when compared with CHO alone [44], the present study observed that CAF + CHO ingestion does not benefit repeated sprint performance versus CAF + PLA, PLA + CHO, or PLA + PLA. By contrast, the total work in PLA + CHO condition increased significantly at Set 3, compared to the CAF + CHO and CAF + PLA conditions. Therefore, it is tempting to speculate that combining CAF with CHO supplementation has no additive effect on prolonged repeated sprint exercise, composed of 10 sets, 5 × 4-s sprints with 20-s rest interval between each sprint. Furthermore, a performance-enhancing effect of CHO seemed to be negated by CAF when recreational male athletes performed 20-kilometer time trial [29]. This apparent discrepancy may be attributed to type (that is, prolonged repeated sprint exercises) and intensity (i.e. high-intensity and short recovery interval) of exercise performed in the present study, because previous study has indicated that anaerobic glycolysis supplies approximately 40% of the total energy during a single 6-s sprint, with a progressive inhibition of glycolysis and decreased ATP production with subsequent sprints [4]. Data also show that blood lactate concentration was not significantly different at pre-test and Set 1 among treatments, but was significantly higher after CAF + PLA ingestion than PLA + CHO and PLA + PLA during later stages of the RSE. Lee et al. [16] demonstrated a significant increase in blood lactate concentrations and decreased fatigue resistance during the late stage of the RSE after CAF ingestion. By contrast, this study and others show that ingesting CHO does not affect the blood lactate response to sprint exercise [45,46]. This may reflect rapidly increasing anaerobic glycolysis, where lactate is produced when ingesting CAF [47]. CAF may impair performance for this type of exercise due to increased accumulation of by-products of anaerobic metabolism [48], a deficiency in the phosphagen system [4], and blocking CNS adenosine receptors [49] or activating Na^+^/K^+^ ATPase [15]. Nevertheless, studies focused on the exact mechanism related with the effects of caffeine on energy substrate or nervous system should be conducted in future.

The present study showed that repeated sprint performance was improved followed CHO ingestion rather than CAF + CHO ingestion or CAF ingestion alone. CHO supplementation before team sport exercise has been demonstrated to significantly improve high-intensity intermittent sprint performance [6] in non-glycogen depleted subjects, which may be attributed to improved cerebral glucose uptake [9], greater CNS function [50], and motor control [45]. Despite the intensity of RSE being higher than ISE [3], CHO ingestion affects the metabolic response to team sport exercise, with a significant increase in glucose concentration found throughout exercise [5,51]. The mechanisms driving this increased blood glucose concentration are largely unknown. Blood glucose concentration initially increases after ingesting CAF + CHO or PLA + CHO and it may be suppressed by endogenous glucose production [52]. The blood glucose levels gradually decreased in the PLA + CHO trial during the RSE, suggesting that intense sprint exercise increases fuel requirements in working muscles and obligates more blood glucose to muscle cells during the RSE. By contrast, the CAF + CHO exhibited higher blood glucose levels during the RSE, partly because caffeine is crucial for maintaining blood glucose concentration by enhancing glycolytic turnover [11].

Although the exact mechanisms of carbohydrate ingestion on exercise performance, especially for exercise duration less than 1 hour, are not well understood, two major explanations are commonly used to interpret the possible ergogenic effects of carbohydrate. Firstly, the general metabolic response to prolonged intermittent exercise with CHO administration is an increase in plasma glucose concentration and higher rates of glucose oxidation during the later exercise stage [9]. Secondly, the presence of carbohydrate in the mouth has been shown to stimulate the receptors in the oral cavity, thus activating specific areas of the brain associated with reward and the regulation of motor activity [27].

CHO ingestion may increase blood glucose concentrations, however, it should be noted that the improved performance in previous studies [45] might be attributed to the glycogen-depleted state prior to the intermittent sprint exercise. In this study, we asked participants to consume a standardized meal 2 hours before exercise test to mimic the real-life situation, e.g., fed athletes before competition, in each trial. The results indicate that ingestion of PLA + CHO provided a small but significant benefit on RSE performance in female athletes. Nevertheless, Colombani et al. [53] reported that CHO administration might not induce performance improvements in male athletes during exercise lasting less than 70-min in postprandial state.

The increases in blood glucose levels and repeated sprint performance induced by CHO ingestion may also involve the central governor. Gastric empty rate of a CHO drink could be slowed by the hypertonic drink [54] and high-intensity intermittent sprint [55]. Jeukendrup et al. [56] reported that CHO ingestion has no effects on exogenous glucose uptake and total CHO oxidation during short-term (~1 hour) high-intensity cycling exercise. Although the mechanism responsible for the improvement in short-term (<1 hour) high-intensity exercise performance with CHO ingestion is not well known, some studies suggest that the CHO mouth rinsing stimulates the receptors in mouth, which modulate central pathways associated with motivation and improve the perceptions of effort [8,27]. Total RPE scores in CAF + CHO and PLA + CHO were slightly with non-significantly lower than those in other treatments (CAF + PLA vs. CAF + CHO vs. PLA + CHO vs. PLA + PLA, 157 ± 18 vs. 152 ± 16 vs. 154 ± 13 vs. 156 ± 17, *p* > .05). More than half of participants in CAF + CHO (7/11, 64%) and PLA + CHO (6/11, 55%) had lesser total RPE scores while comparing with PLA + PLA condition. Therefore, our study might provide some supports for the attenuation of perceptions of effort resulted from the CHO supplementation. In addition, our results in RSE performance are partially in agreement with Beaven et al. [27], who found the CAF and (or) CHO mouth rinse can rapidly enhance initial cycle sprint power production; however, recent study [57] reported that the CHO mouth rinse could not improve performance during simulated team-sport exercise (i.e., Loughborough Intermittent Shuttle Test). Therefore, further studies are needed to clarify the existence of CHO receptors in oral cavity and their effect on RSE performance.

Testosterone and cortisol concentrations have been reported to increase in response to high-intensity activity in humans [58], and with CAF [33] or CHO ingestion [36], respectively. Data from this study show that ingesting CAF or CHO does not alter the circulating levels of testosterone or cortisol, but these levels increased distinctly after the *AT- test* in all four conditions (Figure 6). One study examined alterations in salivary testosterone and cortisol in nine male cyclists completing repeated sprint test (4 sets of 5 × 30-s sprints, interspersed with 30-s recovery intervals) following caffeinated chewing gum ingestion [18]. Results showed that cortisol was increased by 12% and testosterone decreased by 21% compared to placebo condition, although testosterone and cortisol levels were not significantly different between caffeine and placebo trials (*p* > .05). Testosterone concentration is related to exercise intensity and increases with greater force production, and testosterone/cortisol ratio is associated with the anabolic or catabolic status of skeletal muscle during exercise [58]. Cortisol exhibits catabolic functions and increases in volume with repetitive high-intensity exercise, and the rest interval length also affects the acute cortisol response [58]. However, Beaven et al. [34] indicated that the anabolic effect of the increase in testosterone concentrations after CAF ingestion may be counteracted by the opposing catabolic effects of the increase in cortisol concentrations. Walker et al. [35] reported that ingesting CHO produced lower plasma cortisol concentrations than CAF and PLA after cycling for 2 h at 65% VO_2max_, but the type of exercise was different than that used in this study. In addition, plasma cortisol concentrations (approximately 145–193 ng · dL^−1^) induced by the prolonged submaximal exercise in the study of Walker et al. [35] are obviously lower than those in our study. Pre and post-intermittent exercise did not produce significantly different salivary cortisol concentrations after CHO beverage ingestion [59]. According to the results from the current investigation, adding CHO to a solution and ingesting a CAF capsule does not affect hormone variables. This is probably because the intensity of the RSE exerts a strong influence on hormones without ergogenic aids. Changes in these hormones during RSE after ingesting CAF and CHO require further investigation.

## Conclusions

The data demonstrate that ingesting CAF and CHO or only CAF does not increase peak or mean power, or total work during RSE, or improve agility, compared to ingesting PLA + PLA. In contrast to CAF + CHO, CAF + PLA, and PLA + PLA conditions, ingesting PLA + CHO increased sprint performance during 10 sets of 5 × 4-s sprints, with a 20-s rest interval between each sprint (2-min rest between each set). Ingesting PLA + CHO did not alter RPE, agility performance, or hormone profiles. The results suggest that in female athletes, ingesting CHO without CAF before exercise may increase repeated sprint performance.

## Abbreviations

CAF: Caffeine; CHO: Carbohydrate; CI: Confidence interval; CNS: Central nervous system; CV: Coefficient of variation; HR: Heart rate; ICC: Intra-class correlation coefficient; ISE: Intermittent sprint exercise; PLA: Placebo; RPE: Rating of perceived exertion; RSE: Repeated sprint exercise.

## Competing interests

The authors declare that they have no competing of interest.

## Authors’ contributions

CLL and CFC developed the study design, data collection, statistical analysis, and all sport drink tested. TA helped draft the manuscript. JCL was in charge of participant recruitment and management. HWH contributed to the data collection and analysis. WDC provided consultation. All authors contributed to drafting of the manuscript. All authors have read and approved the final manuscript.
